# Alteration of gastric microbiota and transcriptome in a rat with gastric intestinal metaplasia induced by deoxycholic acid

**DOI:** 10.3389/fmicb.2023.1160821

**Published:** 2023-05-03

**Authors:** Zijing Xu, Ling Xiao, Shuaishuai Wang, Yuqin Cheng, Jianping Wu, Yufen Meng, Kaifan Bao, Junfeng Zhang, Chun Cheng

**Affiliations:** ^1^School of Medicine & Holistic Integrative Medicine, Nanjing University of Chinese Medicine, Nanjing, Jiangsu, China; ^2^Laboratory Animal Center, Nanjing University of Chinese Medicine, Nanjing, Jiangsu, China

**Keywords:** gastric intestinal metaplasia, microbiota, bile acids, transcriptome, correlation analysis

## Abstract

**Objective:**

Bile reflux plays a key role in the development of gastric intestinal metaplasia (GIM), an independent risk factor of gastric cancer. Here, we aimed to explore the biological mechanism of GIM induced by bile reflux in a rat model.

**Methods:**

Rats were treated with 2% sodium salicylate and allowed to freely drink 20 mmol/L sodium deoxycholate for 12 weeks, and GIM was confirmed by histopathological analysis. Gastric microbiota was profiled according to the 16S rDNA V3–V4 region, gastric transcriptome was sequenced, and serum bile acids (BAs) were analyzed by targeted metabolomics. Spearman's correlation analysis was used in constructing the network among gastric microbiota, serum BAs, and gene profiles. Real-time polymerase chain reaction (RT-PCR) measured the expression levels of nine genes in the gastric transcriptome.

**Results:**

In the stomach, deoxycholic acid (DCA) decreased the microbial diversity but promoted the abundances of several bacterial genera, such as *Limosilactobacillus, Burkholderia–Caballeronia–Paraburkholderia*, and *Rikenellaceae RC9 gut group*. Gastric transcriptome showed that the genes enriched in gastric acid secretion were significantly downregulated, whereas the genes enriched in fat digestion and absorption were obviously upregulated in GIM rats. The GIM rats had four promoted serum BAs, namely cholic acid (CA), DCA, taurocholic acid, and taurodeoxycholic acid. Further correlation analysis showed that the *Rikenellaceae RC9 gut group* was significantly positively correlated with DCA and RGD1311575 (capping protein-inhibiting regulator of actin dynamics), and RGD1311575 was positively correlated with Fabp1 (fatty acid-binding protein, liver), a key gene involved in fat digestion and absorption. Finally, the upregulated expression of Dgat1 (diacylglycerol acyltransferase 1) and Fabp1 related to fat digestion and absorption was identified by RT-PCR and IHC.

**Conclusion:**

DCA-induced GIM enhanced gastric fat digestion and absorption function and impaired gastric acid secretion function. The DCA–*Rikenellaceae RC9 gut group*–RGD1311575/Fabp1 axis might play a key role in the mechanism of bile reflux-related GIM.

## 1. Introduction

Gastric cancer (GC) is the fifth most common cancer and the fourth leading cause of cancer-related deaths worldwide (Sung et al., 2021), and about half of the global GC cases and deaths occur in China (Nie et al., 2017). Lauren's classification of GC consists of intestinal, diffuse, and mixed types according to the histological structures and biological characteristics. Correa's cascade hypothesis holds that the most common intestinal-type gastric cancer gradually develops from chronic gastritis (CG) to atrophic gastritis, intestinal metaplasia, and dysplasia. Gastric intestinal metaplasia (GIM) has been recognized as a typical precancerous lesion of GC, and a typical pathological feature of GIM is that normal gastric mucosa epithelial cells are replaced by Paneth, goblet, and absorptive cells (Gullo et al., 2020). Substantial epidemiologic evidence has indicated that GIM is the result of the comprehensive effects of multiple environmental factors, such as *Helicobacter pylori* (Hp) infection, bile reflux, aging, race, and lifestyle. Many epidemiologic studies established a close relationship between long-term bile reflux and GIM risk (Hegyi et al., 2018). Zhang et al. observed 668 patients with CG and found that bile reflux is an independent risk factor for gastric precancerous lesions (Zhang et al., 2021a), suggesting that bile reflux inducing the chronic inflammation of gastric mucosa is a critical factor for the development of GIM, but the detailed mechanism is still unclear.

When bile reflux occurs, phospholipase A in pancreatic juice can convert bile lecithin to lysolecithin, which further dissolves the phospholipid layer of the gastric mucosal epithelial cell membrane, enhances cell permeability, and promotes gastric acid to damage gastric mucosa (He et al., 2022b). In addition, bile reflux promotes the reverse diffusion of gastric acid in the gastric mucosa, and gastric acid can stimulate submucosal mast cells to release histamine, which conversely promoted the secretion of gastric acid and pepsin, and finally aggravates mucosal injury (Bechi et al., 1993). Bile acids (BAs) include free and conjugated types. Free BAs consist of cholic acid (CA), deoxycholic acid (DCA), chenodeoxycholic acid (CDCA), and lithocholic acid (LCA), whereas conjugated BAs are conjugated free BAs with glycine or taurine. When bile reflux occurs, the stomach has a higher pH value, and more conjugated BAs accumulate in the stomach, resulting in the continuous damage of gastric epithelial cells (Zhao et al., 2020; Guzior and Quinn, 2021). Previous evidence has revealed that caudal type homeobox 2 (CDX2), SRY-related HMG box-2 (SOX2), and farnesoid X receptor (FXR) are involved in the bile reflux-induced GIM (He et al., 2022b). Yu et al. (2019) found that CDCA and DCA can induce GIM by upregulating CDX2 and mucin-2 (MUC2) expression and the FXR/NF-KB signaling pathway. Yuan et al. (2019) found that DCA can induce gastric epithelial cells and GC cells to express miR-21, which can suppress SOX2 expression and simultaneously induce CDX2 expression. Furthermore, DCA can promote the transcriptional activity of CDX2 and expression levels of GIM-specific genes, including Kruppel-like factor 4, hepatocyte nuclear factor 4 alpha (HNF4α), and cadherin-17 (CDH17). An *in vitro* experiment confirmed that BAs can increase the expression of FXR and several intestinal markers in gastric cells *via* the FXR/SNAI2/miR-1 axis in the stomach (Wang et al., 2021). DCA can promote the expression of histone deacetylase 6 but inhibits the expression of forkhead box protein 3 (FOXP3) in gastric mucosal epithelial cells. FOXP3 can transcriptionally inhibit HNF4α and the expression of downstream intestinal biomarkers (CDX2 and MUC2), suggesting that DCA promotes GIM through epigenetic regulation (Zhang et al., 2022). These results indicated that unknown molecular mechanisms underlie bile reflux-induced GIM.

Owing to rapid developments in non-culture-dependent high-throughput sequencing techniques, evidence shows that gastric microbiota is linked to CG, GIM, and GC. Wang et al. found that the gastric microbial richness dropped progressively from CG, intestinal metaplasia (IM), and intraepithelial neoplasia (IN) to GC in a cohort of 102 patients, and three bacterial phyla (Actinobacteria, Bacteroidetes, and Firmicutes) were enriched in IN and GC (Wang et al., 2020b). In normal gastric juice, the conjugated and free BAs are almost equivalent; however, Zhao et al. (2020) found that conjugated BAs significantly increased in the stomach of patients with bile reflux, which might be the original driving force that alters the gastric microbial microbiota. Wang et al. (2022) found that conjugated BAs significantly increased with the abundances of several lipopolysaccharide-producing bacteria, such as *Prevotella melaninogenica, Neisseria sicca*, and *Veillonella parvula*, in the gastric juices of patients with gastritis and bile reflux and patients with GC. Surprisingly, the abundance of lipopolysaccharide-producing bacteria is positively correlated with the level of taurodeoxycholic acid (TDCA) in gastric juices. These results manifested that the interaction between gastric flora and BAs plays a key role in the development of bile reflux-related GIM.

Many reports have shown that several procedures can simulate bile reflux to induce gastric precancerous lesions in rat and mouse models. Previous reports successfully replicated a rat model with chronic atrophic gastritis (CAG) by combining hunger–satiety disorders and alternated gavage of 0.1% ammonia and 20 mmol/L DCA in 10th to 12th weeks; they found that the Chinese medical formula [Huangqi Jianzhong decoction (Liu et al., 2018)] and one of its herbs [Huangqi, *Astragalus membranaceus* (Fisch.) Bunge (Liu et al., 2022a)] can ameliorate CAG lesions by altering gut microbiome and metabolism. Wang et al. (2006) successfully replicated the rat model of CAG by administrating 2% sodium salicylate solution and freely drinking 20 mmol/L sodium deoxycholate solution for 6 weeks. These experiences laid the solid foundation for imitating bile reflux-induced GIM and exploring potential etiological mechanisms. Thus, we established a rat model of GIM by administrating 2% sodium salicylate solution and allowing the rats to freely drink 20 mmol/L sodium deoxycholate solution for 12 weeks; and found that the DCA–*Rikenellaceae RC9 gut group*–RGD1311575/Fabp1 axis might be the key biological mechanism during the development of bile reflux-induced GIM by integrating the microbiome, transcriptome, and targeted metabolomics of serum BAs.

## 2. Materials and methods

### 2.1. Inducement of rat with gastric intestinal metaplasia

Specific-pathogen-free (SPF) male Sprague–Dawley (SD) rats (170 ± 20 g, 5–6 weeks old) were purchased from Zhejiang Weitong Lihua Experimental Animal Technology Co., Ltd. (Zhejiang, China). All rats were subjected to 12 h light/12 h dark cycles, controlled ambient room temperature (20 ± 2°C), and air humidity (50–70%). The study protocol was approved by the Animal Ethics and Protection Committee of Nanjing University of Chinese Medicine (Nanjing, China; No. 202102A004). All the SD rats were fed with SPF-grade sterilized diet and water. After 7 days of adaptive feeding, the animals were randomly assigned to either the control or model group. The model rats were treated with an improved method according to the previous literature (Wang et al., 2006), freely drank 20 mmol/L of deoxycholate sodium every day (Beyotime Biotech. Inc., China, No. ST2049), and received 2 mL of 2% sodium salicylate (Sigma, No. S3007) every morning. The control rats were fed with granular SPF-grade fodder and freely drank water and received 2 mL of sterile water every day. General information was collected, including food intake, water consumption, weight, and stool traits. After 12 weeks, GIM was successfully observed in the gastric antrum tissues through pathological examination.

### 2.2. Sample collection and processing

All rats were fasted for 24 h without water deprivation. After the administration of isoflurane, the rats were killed by exsanguination. The blood was collected, and the serum was centrifuged at 2,500 rpm for 25 min and stored at −80°C after being quickly frozen in liquid nitrogen. The rat stomachs were incised along the greater curvature, cleaned, and washed gently with normal saline water. According to the consensus of diagnosis and management of atrophic gastritis (Shah et al., 2021), three adjacent strips of gastric antrum tissues were collected, one sample was fixed in 4% neutral paraformaldehyde and embedded by paraffin within 48 h, and the other two samples were immediately frozen in liquid nitrogen for microbial profiling and transcriptome sequencing, respectively.

### 2.3. Histopathological examination

The paraffin-embedded gastric mucosa tissues were cut into 5 μm sections. After routine dewaxing and hydration, hematoxylin and eosin (H&E) staining was performed using an H&E Staining Kit (Beijing Solarbio Science and Technology Co., Ltd, G1120-100). The H&E staining steps were as follows: hematoxylin staining for 3 min, immersion in differentiation for 3 min, eosin staining for 30s, ethanol gradient dehydration, xylene transparent application, and sealing with neutral gum. An Alcian Blue–periodic acid Schiff (AB–PAS) staining kit (Beijing Solarbio Science and Technology Co., Ltd., G1285-50) was used in evaluating the status of GIM, and the routine procedure was as follows: Alcian blue staining for 10 min, oxidization with an oxidizer for 5 min, immersion in Schiff reagent for 10 min, washing with flowing water for 10 min, hematoxylin staining for 1–2 min, immersion in acidic differentiation solution for 5 s, staining in Scott's solution, dehydration with ethanol, hyalinization with xylene, and final sealing with neutral gum. The degrees of gastric atrophy and IM were assessed under a microscope.

Paraffin-embedded gastric antra were cut into serial sections (~5μm) for the immunohistochemistry (IHC) staining of FABP1 and diacylglycerol acyltransferase 1 (DGAT1), and a standard protocol was used (Yang et al., 2018). Anti-FABP1 and anti-DGAT1 were used in staining the GIM cells. Gastric antral tissue sections were removed from the paraffin-embedded tissues, dewaxed with xylene, rehydrated with graded ethanol, and heated in citrate antigen repair buffer (G202, pH 6.0) for 20 min for antigen repair. The endogenous peroxidase activity was quenched with 3% hydrogen peroxide for 30 min, and then, the slides were blocked by 3% BSA for 30 min to avoid non-specific staining. The cells were then incubated with polyclonal antibodies against FABP1 (ab-171739, 1:3000 dilution; Abcam, Cambridge, United Kingdom) and DGAT1 (1:200; Proteintech, China) overnight at 4°C, removed from the refrigerator at 4°C, washed three times with PBS, and then incubated with the appropriate amount of secondary antibody at 37 °C for 50 min. Finally, the slices were stained with diamino-benzidine (DAB) and counterstained with hematoxylin. After dehydration, the slices were sealed and examined under the microscope.

### 2.4. Analysis of gastric mucosa microbiota

#### 2.4.1. DNA extraction, PCR amplification, and gene sequencing of gastric mucosa

The gastric microbiota was detected by Shanghai Biozeron Biotechnology Co., Ltd., according to the previously reported method (Kang et al., 2022). The total DNA of gastric mucosa was extracted, the concentration and purity of DNA were detected by 1% agarose gel electrophoresis, the total DNA was used as a template for the specific amplification of bacterial 16S rDNA V3–V4 regions, and the primers were 341F 5′-CCTAYGGGRBGCASCAG-3′ and 806R 5′-GGACTACNNGGGTATCTAAT-3′. Polymerase chain reaction (PCR) products were retrieved as the MiSeq PE library, and double terminal sequencing was performed using the Illumina PE250 platform.

#### 2.4.2. Bioinformatics analysis

The raw data were filtered by QIIME (version 1.8.0) software. The process involves primer removal, quality filtering, denoise, and de-chimerism. The obtained clean reads were clustered to generate operational taxonomic units (OTUs) at a similarity cutoff value of 97% with Usearch (http://www.drive5.com/usearch/), and OTUs with the highest frequency were selected as the representative sequences. The representative OTUs were annotated with responding taxon at six levels (phylum, class, order, family, genus, and species) by the RDP Classifier Naive Bayes algorithm. Microbial richness and diversity were evaluated through alpha diversity analysis, and the bacterial richness indices (observed OTUs, Ace, and Chao) and diversity indices (Shannon and Simpson) were calculated using QIIME (version 1.8.0). Potential bacterial taxa were screened through linear discriminant analysis effect size, and the Kruskal–Wallis test was performed. A linear discriminant analysis (LDA) can estimate the impact of the abundance of each bacterial taxon on the effect of difference. The PICRUSt package (Langille et al., 2013) is a common bioinformatics tool for predicting the functional abundance of sequencing 16S rDNA, and the Kyoto Encyclopedia of Genes and Genomes (KEGG) and Clusters of Orthologous Groups (COG) functions were predicted in the development of GIM.

### 2.5. Transcriptome analysis of gastric mucosa

Total RNA of gastric mucosa was extracted according to the standard protocol of the TRIzol Kit (Invitrogen, CA, USA). The purity of RNA was detected by a Nanodrop 2000 Ultrafine Spectrophotometer (Thermo Fisher Scientific, USA), and the integrity of RNA was measured with an Agilent 2100 Bioanalyzer (Agilent, USA). mRNA was enriched and reversed into cDNA with TruSeq^TM^ RNA Sample Prep Kit (Illumina, USA), and cDNA was subjected to end repair. The cDNA library was amplified by bridge PCR, and high-throughput sequencing was performed using the Illumina NovaSeq 6000 platform.

In the RNA-seq analysis, gene expression levels were estimated by the number of clean reads mapped to genomic regions. According to the comparison of all samples to reference genomes, the value of fragments per kilobase of the exon model per million mapped fragments was calculated as the expression amount of a gene or transcript. Differentially expressed genes (DEGs) were screened by a difference significance analysis and were filtered according to the criteria of *FDR* ≤ 0.05 and the absolute value of log_2_ (fold change) ≥ 1. Then, the volcano map presented the whole status of gene differential expression. Finally, the KEGG pathway and GO biological process enrichment analyses were performed and visualized using the “clusterProfiler” R package (Yu et al., 2019) for the prediction of DEG-related signaling pathways.

### 2.6. Targeted metabolomics analysis of serum bile acids

The targeted metabolomics of serum BAs was analyzed according to the previous reports (Zhao et al., 2020; Xie et al., 2021). The method was briefly described as follows: First, 33 kinds of BA standards were accurately weighed, and the mother liquor contained 25 μg/mL standard of each BA and was diluted to the linear working solutions, including 25,000, 15,000, 5,000, 2,500, 5,00, 250, 50, 25, 15, 5, 2.5, and 1.5 ng/mL methanol. The 33 BA standards included CA, CDCA, alpha-muricholic acid (α-MCA), beta-muricholic acid (β-MCA), TCA, taurochenodeoxycholic acid (TCDCA), tauro-alpha-muricholic acid (T-α-MCA), glycocholic acid (GCA), glycochenodeoxycholic acid (GCDCA), DCA, LCA, ursodeoxycholic acid (UDCA), 12-ketolithocholic acid (12-ketoLCA), TDCA, glycodeoxycholic acid (GDCA), glycoursodeoxycholic acid (GUDCA), tauroursodeoxycholic acid (TUDCA), taurohyodeoxycholic acid (THDCA), taurohyocholic acid (THCA), hyodeoxycholic acid (HDCA), 23-nordeoxycholic acid (NorDCA), allolithocholic acid (alloLCA), 3-dehydrocholic acid (3-DHCA), cholic acid-d4 (CA-d4), lithocholic acid-2,2,4,4-d4 (LCA-d4), ursodeoxycholic acid-2,2,4,4-d4 (UDCA-d4), glycocholic acid-2,2,4,4-d4 (GCA-d4), glycochenodeoxycholic acid-2,2,4,4-d4 (GCDCA-d4), and glycodeoxycholic acid-2,2,4,4-d4 (GDCA-d4). All the BAs were obtained from Steraloids (United States).

GCA-d4, LCA-d4, CDCA-d4, UDCA-d4, CA-d4, and GCDCA-d4 solutions at specific concentrations were blended as internal standards. Second, the serum remained in equilibrium at room temperature for 2 h and was mixed well by vortexing. After centrifugation of 4,000 rpm for 10 min at 4°C, the centrifugal supernatant was taken and diluted 10 and 1,000 times, respectively. Third, 100 μL of dilution was mixed with 500 μL of acetonitrile/methanol mixture (8:2) containing internal standards and centrifuged at 12,000 rpm for 20 min for protein denaturation and removal. Finally, the supernatant was dried up by a nitrogen blower and redissolved in a solution containing 100 μL of water/acetonitrile (8:2) and formic acid (0.1%). After centrifugation, 2 μL of supernatant was injected into ultrahigh-performance liquid chromatography–tandem mass spectrometry (UHPLC–MS/MS) system for targeted metabolic analysis.

The UHPLC–MS/MS system included AB Sciex QTRAP 6500+ mass spectrometer and AB Scienx ExionLC^TM^ AD liquid chromatography. Separation was performed on a Waters ACQUITY UPLC BEH C18 column (2.1 mm × 100 mm, 1.7 μm) at 50°C. The mobile phase, consisting of 0.1% formic acid in water (solvent A) and acetonitrile (solvent B), was delivered at a flow rate of 0.30 mL/min. The solvent gradient was set as follows: initial 20% B, 0.5 min; 20–35% B, 1 min; 35–37% B, 2.5 min; 37–38% B, 4.1 min; 38–39% B, 6 min; 39–40% B, 6.5 min; 40–44% B, 8.5 min; 44–45% B, 9 min; 45–52% B, 9.5 min; 52–65% B, 12.5 min; 65–100% B, 13 min; 100–20% B, 15.1 min; and 20% B, 17 min. The BAs eluted from the chromatographic column were ionized by electrospray ionization in negative-ion mode. The parameters were as follows: ion source temperature (550°C), ion spray voltage (−4500 V), curtain gas (30 psi), and ion source gas of 1 and 2 (65 psi). The mass spectrometer was operated in negative multiple reaction mode. Quantitative metabolite data were obtained, and multivariate pattern recognition analysis and difference analysis were carried out.

### 2.7. RT-PCR verification

The total RNA of gastric mucosa was extracted with TRI Reagent (Sigma, St. Louis, USA), cDNA was obtained by reverse transcription of 5 μg of total RNA, and cDNA was retrieved and purified by 1.5% agarose gel electrophoresis. Ten pairs of primers (Supplementary Table 1) were designed for fluorescent quantitative PCR using SYBR Green or TaqMan (Applied Biosystems, Foster City, USA) kits, and the PCR protocol was as follows: initial denaturation at 95°C for 10 min, followed by 40 cycles of denaturation at 95°C for 5 s and annealing at 60°C for 60 s. The reaction conditions recommended GoTaq Probe qPCR Master Mix (Promega, Madison, WI, USA) or SYBR Green/ROX qPCR Master Mix (Thermo Fisher Scientific, Waltham, MA, USA). The expression level of β-actin as the internal control was determined. The experiment was repeated three times, and the 2^−Δ*ΔCt*^ method was used in calculating the levels of gene expression.

### 2.8. Statistics

The data were processed using SPSS 26.0 (SPSS, Inc.). The normally distributed metrological data were analyzed by Student's *t*-test and presented as means ± standard deviations (SDs), whereas non-normally distributed metrological data were analyzed by the Mann–Whitney *U* non-parametric test. Two-sided *P*-values of < 0.05 were assumed to be statistically significant.

## 3. Results

### 3.1. General characteristics of gastric mucosa in GIM rats

**Figure 1:**
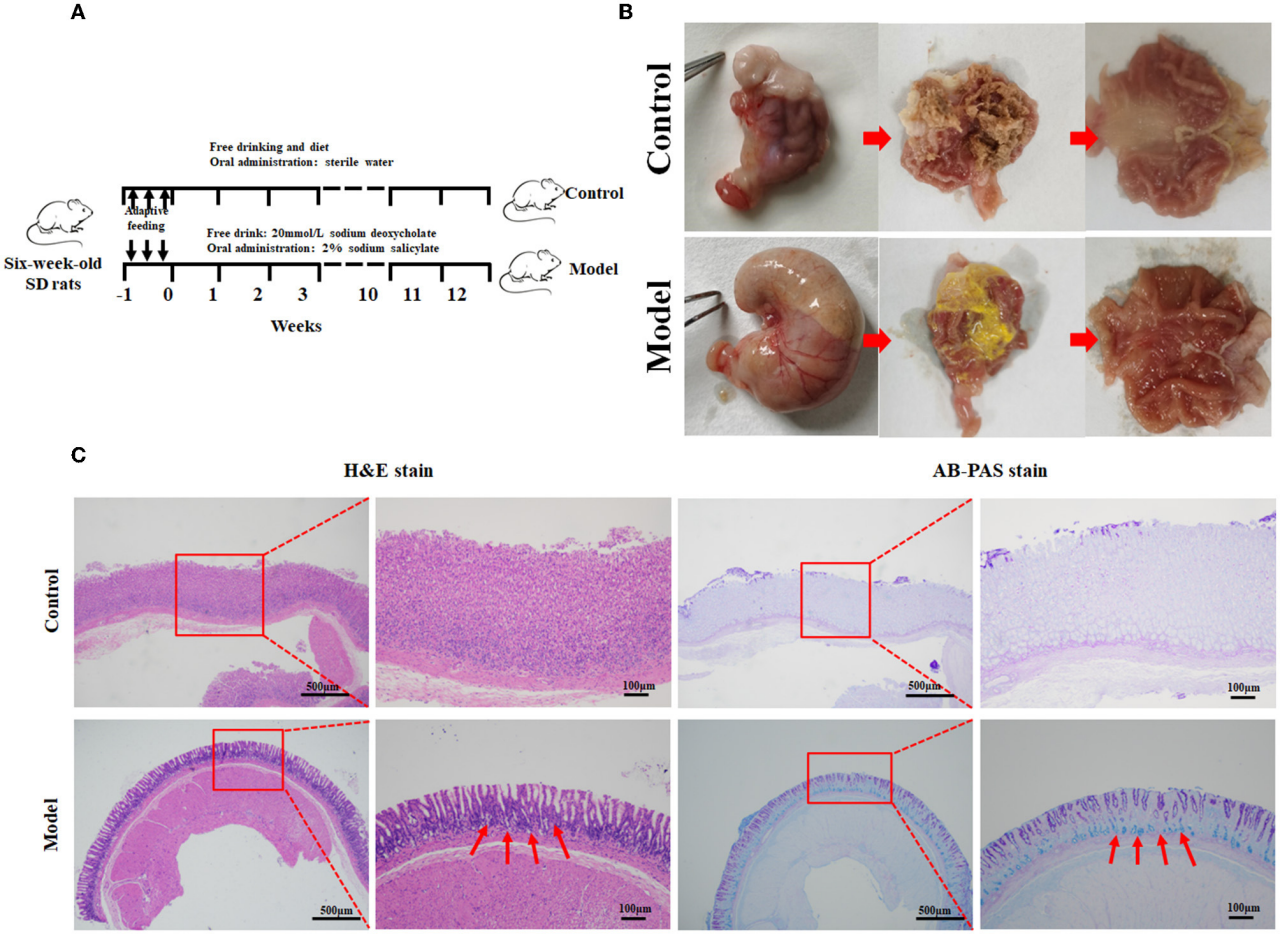
Establishment of the rat model with gastric intestinal metaplasia. **(A)** Flowchart of the model with GIM rats. **(B)** Morphological evaluation of the stomachs. **(C)** Histopathological analysis of gastric mucosa using H&E and AB-PAS staining in the control group (*n* = 10) and model group (*n* = 9). The red arrows refer to the goblet cells.

In this study, the rat model with GIM was satisfactorily established by administrating 2 mL of 2% sodium salicylate, and the rats had free access to 20 mmol/L sodium deoxycholate for 12 weeks (Figure 1). The flowchart is given in Figure 1A. General morphology showed that the gastric mucosae of the control rats were soft, shiny, and moist. The mucosa-suffused rich fold, uniform texture, and brown dry contents were observed. However, the stomachs of model rats were mostly distended, and the gastric mucosae were pale and had less swollen folds commonly covered with yellow and wet contents, consistent with the performance of bile reflux (Li et al., 2020; Figure 1B). The suspicious mucosal lesions of the gastric antrum were obtained for pathological analysis. H&E staining showed that the gastric mucosae of the control rats were intact, and the mucosal epithelial cells were arranged in an orderly manner without infiltration, edema, atrophy, and other abnormal features. The gastric mucosae of the model rats became thinner, and the glands were arranged irregularly, shrank, and reduced. Meanwhile, the gastric fovea lengthened, twisted, and even dendritically extended into the lamina propria. The appearance of goblet cells and the thickened submucosal muscle layer indicated the occurrence of GIM. The characteristic acid mucin secreted from intestinal metagenetic cells was stained blue by AB–PAS staining, and thus, the significantly enhanced blue stain in the gastric mucosa indicated the occurrence of GIM (Figure 1C). The positive rate of the AB–PAS staining of gastric mucosa was 80% (16/20), the occurrence rate of goblet cells with H&E staining was 56.3% (9/16), and the six dipositive samples were collected for the following experiment as the GIM rats (also named the model group).

### 3.2. Alteration of gastric mucosal microbiota in GIM rats

**Figure 2:**
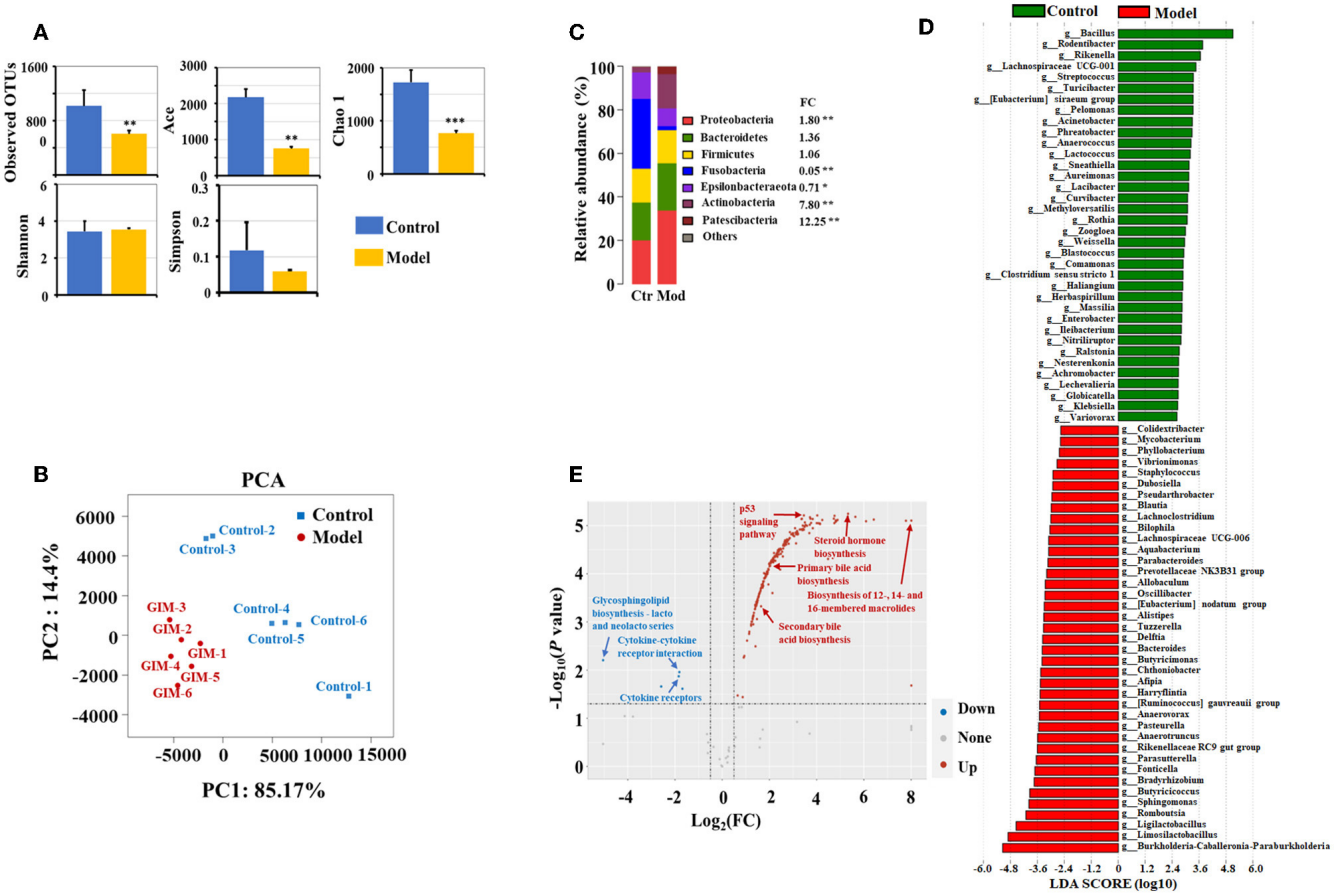
Altered microbiota of gastric mucosa in GIM rats. **(A)** Mann–Whitney *U*-test was conducted to analyze the difference of microbial alpha diversity in the gastric mucosa between the control group (*n* = 6) and model group (*n* = 6), and the double stars mean *P* < 0.01. **(B)** Beta diversity analysis showed a significant difference in the gastric microbiota between the control and model groups. **(C)** The community structure of gastric microbiota at the phylum level. **(D)** LDA analysis presented the potential GIM-related bacterial genera in the gastric mucosa. **(E)** Difference analysis presented the GIM-related KEGG predictive functions based on the gastric mucosal microbiota.

The microbiota obviously changed in the stomachs of the GIM rats induced by 2% sodium salicylate and 20 mmol/L sodium deoxycholate (Figure 2). Compared with the control group, the richness indices (observed OTUs, Ace, and Chao1) of microbiota significantly decreased in the gastric mucosa of the model group (*P* < 0.01), but no differences were observed in the diversity indices (Shannon and Simpson; *P* > 0.05; Figure 2A). A principal component analysis (PCA) was selected for beta diversity analysis, and the results showed that the composition of gastric mucosal microbiota in the model group was distinctly different from that in the control group (Figure 2B). At the phylum level, the dominant phyla in the gastric mucosa included Proteobacteria, Bacteroidetes, Firmicutes, Fusobacteria, Epsilonbacteraeota, Actinobacteria, and Patescibacteria. Compared with the control group, the model group had higher abundances of Proteobacteria (*P* = 0.004), Actinobacteria (*P* = 0.004), and Patescibacteria (*P* = 0.004) but had lower abundances of Fusobacteria (*P* = 0.004) and Epsilonbacteraeota (*P* = 0.010; Figure 2C). LDA screened 203 bacterial taxa that can distinguish the model group from the control group and exhibited the dominant bacterial genera (relative abundance > 0.5%). Among the different genera, 39 genera were enriched in the model group, including *Limosilactobacillus* (33.85%), *Burkholderia–Caballeronia–Paraburkholderia* (28.62%), *Ligilactobacillus* (7.36%), *Romboutsia* (4.01%), *Sphingomonas* (2.13%), and *Bradyrhizobium* (1.09%). Meanwhile, 36 bacterial genera were enriched in the control group, including *Bacillus* (25.12%), *Rodentibacter* (1.74%), *Streptococcus* (0.67%), *Turicibacter* (0.61%), and *Acinetobacter* (0.53%; Figure 2D).

PICRUST was used in predicting the KEGG function according to the gastric mucosal microbiota. Here, we used FDR <0.05 and |log (FC)| ≥ 0.5 as standards to filter the potential predictive function. Compared with the control group, 245 predictive functions significantly increased, and seven predictive functions significantly decreased in the model group (Figure 2E). A small *P*-value indicated a highly predictive function. The most noteworthy signaling pathways included eight upregulated signaling pathways (steroid hormone biosynthesis, fluorobenzoate degradation, p53 signaling pathway, RIG-I-like receptor signaling pathway biosynthesis of 12-, 14-, and 16-membered macrolides, arachidonic acid metabolism, primary bile acid biosynthesis, and secondary bile acid biosynthesis) and three downregulated signaling pathways (bacterial invasion of epithelial cells, glycosaminoglycan biosynthesis chondroitin sulfate, and cytokine receptors).

### 3.3. Significant changes in gene expression profiles in the gastric mucosa of GIM rats

**Figure 3:**
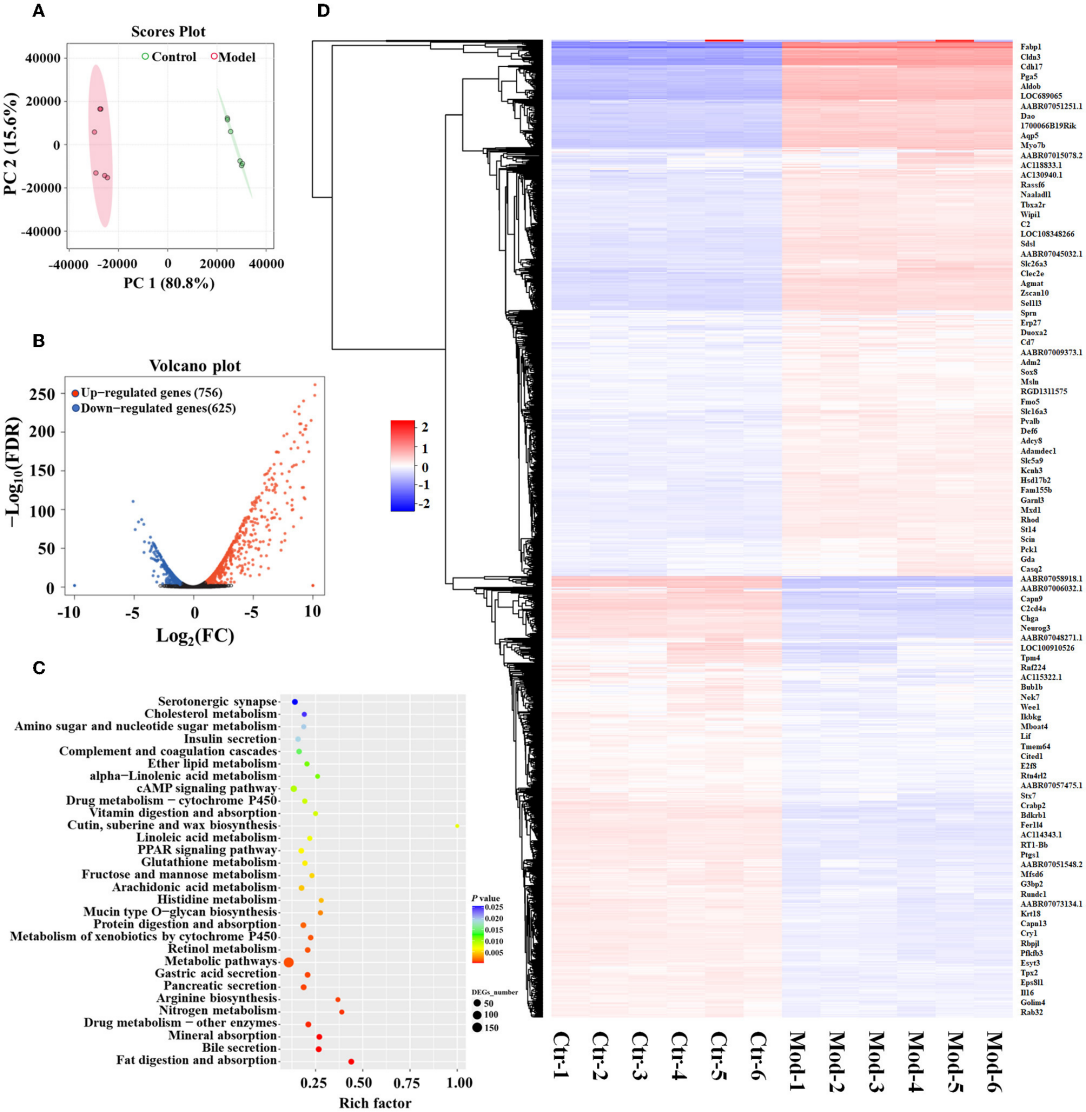
DEGs of GIM-related gastric mucosa and functional prediction. **(A)** Principal component analysis (PCA) of gastric mucosa gene expression profiles in the model and control groups. **(B)** Difference analysis of gastric mucosa gene expression profiles between the model and control groups. **(C)** Hierarchical clustering heatmap of the profile of DEGs between the model and control groups. **(D)** Function enrichment analysis of significant DEGs.

RNA-sequencing analysis demonstrated significant changes in gene expression patterns in the gastric mucosa when GIM occurred (Figure 3). An unsupervised principal component analysis (PCA) revealed the distinct gene expression profiles in the gastric mucosa between the model and control groups (Figure 3A). First, DEGs were screened according to the criteria of a fold difference |log_2_ (FC) | ≥ 1 and FDR <0.05. A total of 756 genes were significantly upregulated, and 625 genes were significantly downregulated in the gastric mucosa of the model group (Figure 3B) compared with the control group. Second, the hierarchical clustering algorithm showed that 99 genes with the most significant differences were associated with GIM occurrence; the significantly upregulated genes included *Fabp1* (fatty acid-binding protein, liver), *Cdh17* (cadherin-17), *Gda* (guanine deaminase), *Hsd17b2* (estradiol 17-beta-dehydrogenase 2), *Pck1* (chain A of phosphoenolpyruvate carboxykinase, cytosolic [GTP]), *Slc26a3* (chloride anion exchanger), and *Aqp5* (aquaporin-5), whereas the gene *RT1-Bb* (Rano class II histocompatibility antigen, B-1 beta chain), which is associated with MHC class II protein complex pathway, was significantly downregulated (Figure 3C). Finally, the mapping of the DEGs to KEGG pathway analysis explored the biological functions of DEGs, and the results showed that the DEGs were mainly enriched in four signaling pathways, including fat digestion and absorption (ko04975), bile secretion (ko04976), pancreatic secretion (ko04972), and gastric acid secretion (ko04971l; Figure 3D). The results suggested that bile reflux can enhance the gastric function of fat digestion and absorption but weaken the gastric function of gastric acid secretion.

### 3.4. Significant alteration of serum bile acids in GIM rats

**Figure 4:**
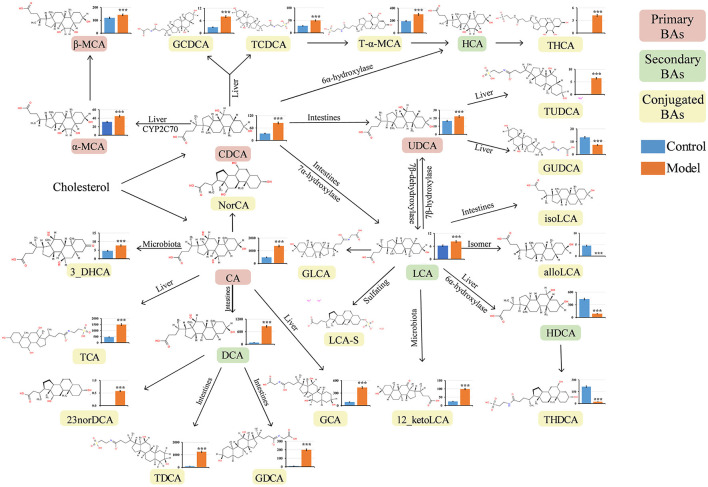
Quantitative analysis of serum bile acids in GIM rats. The Mann–Whitney *U*-test was conducted to present the differences between the control and model groups. *P < 0.05, **P < 0.01, and ***P < 0.001. The vertical axis represents the concentration of serum bile acids (ng/mL). CA, cholic acid; CDCA, chenodeoxycholic acid; α-MCA, alpha-muricholic acid; β-MCA, beta-muricholic acid; GCA, glycocholic acid; GCDCA, glycochenodeoxycholic acid; DCA, deoxycholic acid; LCA, lithocholic acid; TCA, taurocholic acid; TCDCA, taurochenodeoxycholic acid; T-α-MCA, tauro-alpha-muricholic acid; UDCA, ursodeoxycholic acid; 12-ketoLCA, 12-ketolithocholic acid; TDCA, taurodeoxycholic acid; GDCA, glycodeoxycholic acid; GUDCA, glycoursodeoxycholic acid; TUDCA, tauroursodeoxycholic acid; THDCA, taurohyodeoxycholic acid; THCA, taurohyocholic acid; HDCA, hyodeoxycholic acid; NorDCA, 23-Nordeoxycholic acid; alloLCA, allolithocholic acid; 3-DHCA, 3-dehydrocholic acid.

Numerous studies have revealed that bile reflux can induce chronic inflammation in the gastric mucosa and plays a key role in the development of GIM (He et al., 2022b). BAs are the main components of bile and are mainly synthesized in the liver. Under normal conditions, the liver converts cholesterol into primary BAs, and then, the secreted BAs enter the intestinal lumen and are modified by intestinal bacteria to produce many kinds of secondary BAs (Kriaa et al., 2019). BAs are absorbed into the blood through the terminal ileum and circulate back to the liver through a process termed enterohepatic circulation of BAs (di Gregorio et al., 2021). When bile reflux occurs, certain BAs can stimulate gastric mucosa, induce inflammatory lesions, and finally lead to carcinogenesis in the gastric mucosa. In this study, 2% sodium salicylate damaged the gastric mucosa and had a broken window effect, freely drinking 20 mmol/L sodium deoxycholate solution simulated bile reflux, and similar bile reflux was observed in the stomachs of the GIM rats. To explore which BAs affect the occurrence of GIM, we analyzed 33 serum BAs through UHPLC–MS/MS. A total of 23 serum BAs were detected, and their levels significantly varied between the control and model groups (*P* < 0.05). Their transformational relationship is presented in Figure 4 (Wahlström et al., 2016). The observed five primary BAs (CDCA, CA, α-MCA, β-MCA, and UDCA) were all significantly upregulated in the model group. With regard to serum secondary BAs in the GIM rats, DCA and LCA were significantly upregulated, whereas HDCA was significantly downregulated. With regard to 15 serum-conjugated BAs, 12 of them had significantly higher levels in the model group than in the control group, including the glycine or taurine-conjugated BAs, such as GDCA, TDCA, and TCA. Conversely, three conjugated BAs in the model group significantly decreased, including alloLCA, GUDCA, and THDCA.

Interestingly, the primary BAs (CDCA, CA, and UDCA) and the secondary BA LCA significantly increased in the model group, which can be converted to many kinds of conjugated BAs. Here, we used DCA to induce GIM rats, and its derivative BAs (TDCA and GCDA) in the model group were much higher than those in the control group, suggesting that certain bacteria participate in the modification of DCA.

### 3.5. Correlated network of gastric microbiota, bile acids, and gene expression

**Figure 5:**
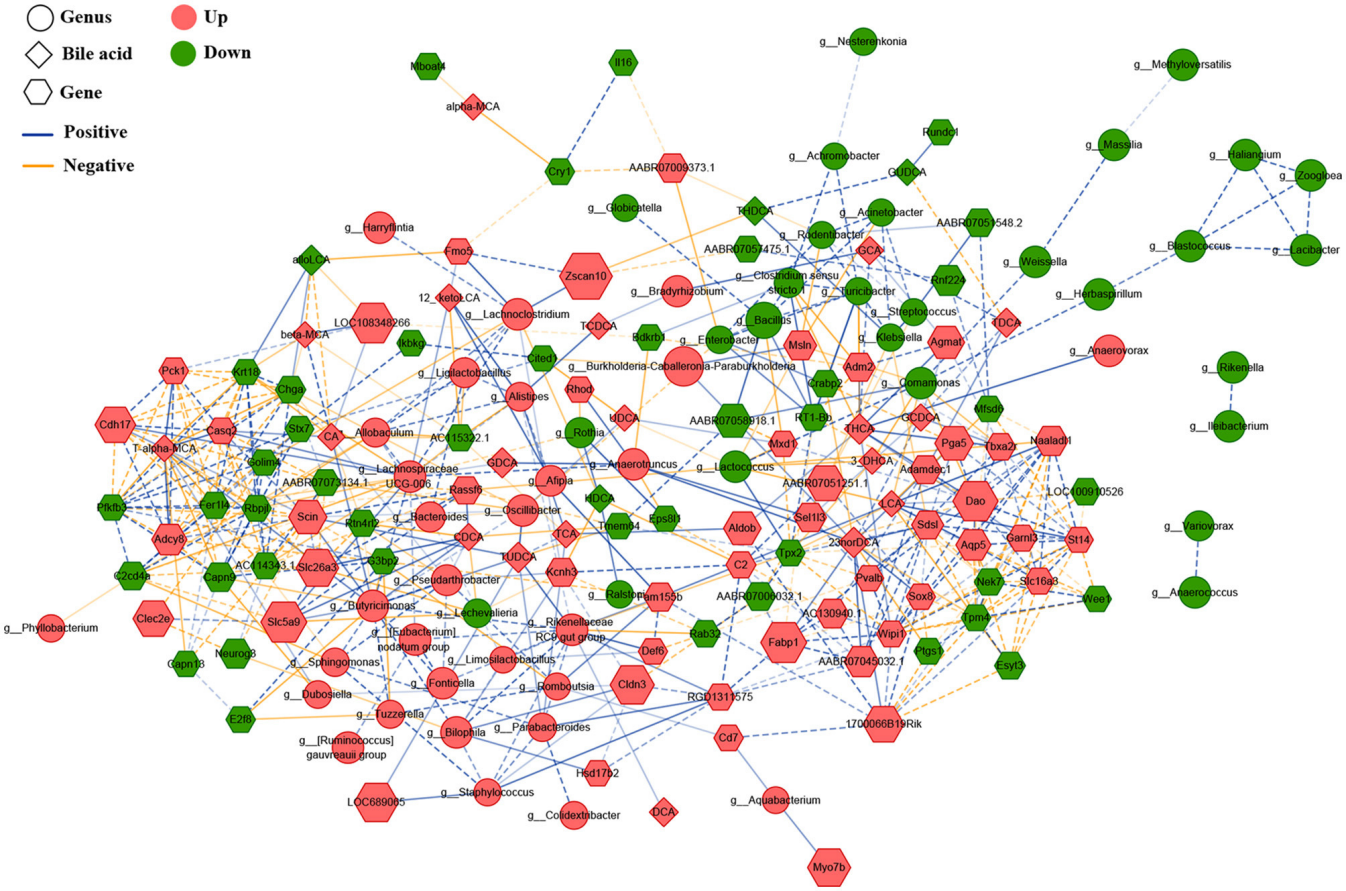
Correlated network of gastric microbiota, bile acids, and expression genes. Here, the relative abundances of the differential bacteria were combined based on LDA, the concentration of serum bile acids, and the levels of differentially expressed genes, and a correlation network was constructed through Spearman's correlation analysis. The round nodes represent the bacterial genera, the diamond nodes represent serum bile acids, and the hexagonal nodes represent the expressed genes in the gastric mucosa. The size of the node represents the quantitative ratio of the model group to the control group. The solid line represents a significant positive correlation (r > 0.9, *P* < 0.01), whereas the dotted line indicates a significant negative correlation (*r* < – 0.9, *P* < 0.01).

To explore underlying interactions among the gastric microbiota, BAs, and gene expression, we performed Spearman's correlation analysis to build a correlation network, including 75 different bacterial genera, 23 serum BAs, and 99 DEGs in the gastric mucosa (Figure 5). First, DCA was significantly positively correlated with the *Rikenellaceae RC9 gut group* (*r* = 0.903), which had a significantly positive correlation with several gastric bacteria, including dominant *Limosilactobacillus* (*r* = 0.911), *Bilophila* (*r* = 0.948), *Alistipes* (*r* = 0.902), *Butyricimonas* (*r* = 0.920), *Fonticella* (*r* = 0.908), *Staphylococcus* (*r* = 0.911), *[Eubacterium] nodatum group* (*r* = 0.913), and *Parabacteroides* (*r* = 0.910). This result indicated that gastric DCA can alter the composition of gastric microbiota. The *Rikenellaceae RC9 gut group* was positively correlated with serum TUDCA (*r* = 0.928), suggesting that the gastric microbiota modifies BAs. In addition, the *Rikenellaceae RC9 gut group* was significantly positively correlated with the expression level of gene capping protein-inhibiting regulator of actin dynamics (RGD1311575; *r* = 0.911), and RGD1311575 was significantly positively correlated with Fabp1 (*r* = 0.930). Hashimoto et al. (2004) found that FABP1 was highly expressed in the GIM tissues (>95%) but had low expression in gastric tubular adenoma (22%). FABP1 had a positive rate of 38% in the GC tissues and had no relationship with the progression, prognosis, and fatty acid synthase status of the carcinoma. The DCA–*Rikenellaceae RC9 gut group*–RGD1311575/Fabp1 axis might be a key controller in the development of bile reflux-induced GIM.

### 3.6. Verification of key signaling pathways related to GIM

**Figure 6:**
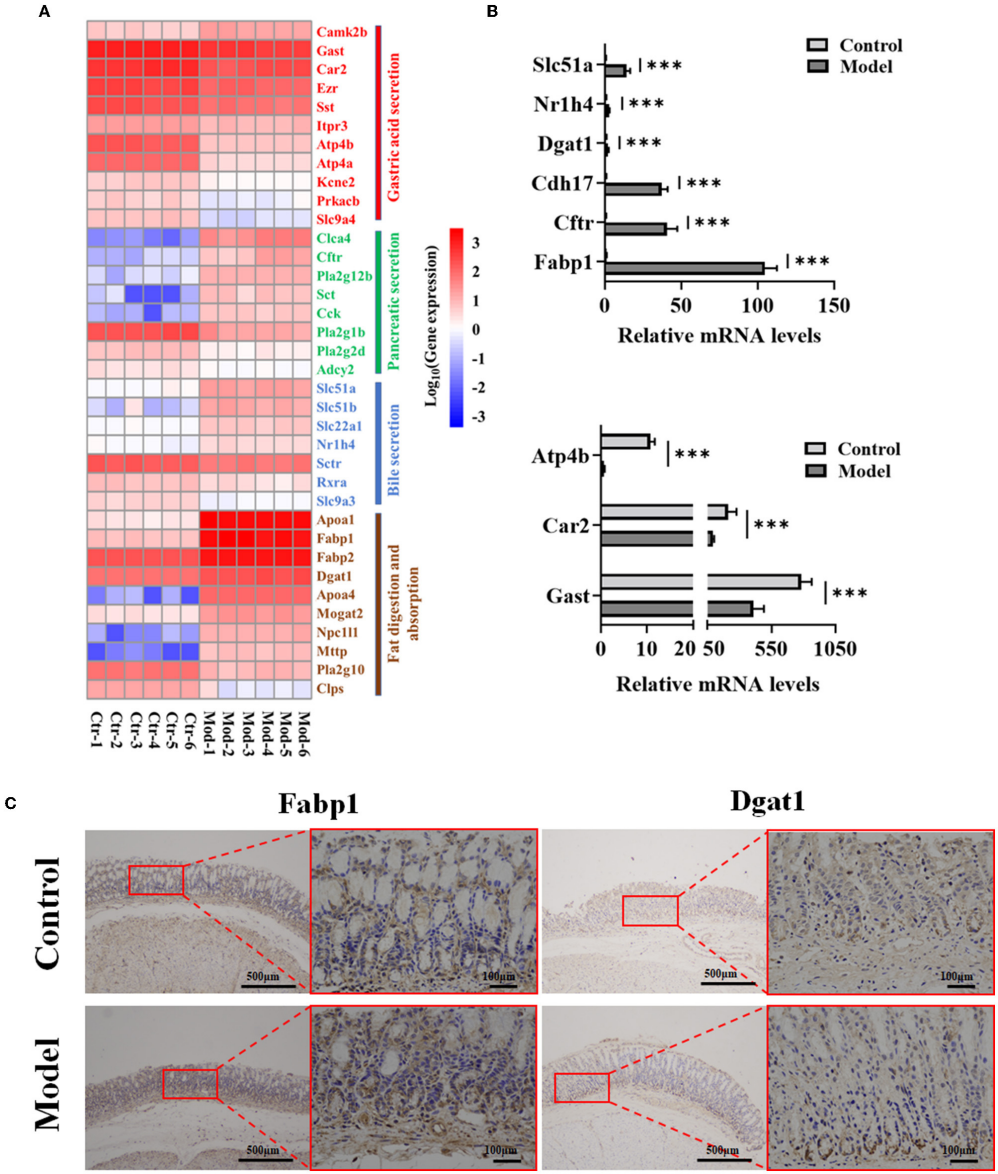
Verification of nine genes in four signaling pathways related to GIM development. **(A)** DEG heatmap of gastric mucosa on the KEGG enrichment pathways. **(B)** Verification of nine genes in gene expression profiles by RT-PCR, ***P < 0.001. **(C)** Validation of Fabp1 and Dgat1 in gastric mucosa tissues by IHC (*n* = 6).

To explore the potential molecular mechanism of bile reflux-induced GIM, we focused on the DEGs participating in the four KEGG pathways, including gastric acid secretion, pancreatic secretion, bile secretion, and fat digestion and absorption (Figure 6A). Spearman's correlation analysis presented the linkage between expression genes and serum BAs (Supplementary Figure 1) and between the expression genes and gastric differential bacteria (Supplementary Figure 2). The results showed that the serum level of DCA was significantly positively correlated with Apoa4 (apolipoprotein A-IV precursor) (*r* = 0.774), Mogat2 (2-acylglycerol O-acyltransferase 2) (*r* = 0.755), Npc1l1 (NPC1-like intracellular cholesterol transporter 1 precursor) (*r* = 0.762), Slc22a1 (solute carrier family 22 member 1) (*r* = 0.776), Slc51a (organic solute transporter subunit alpha) (*r* = 0.755), Cck (cholecystokinin preproprotein) (*r* = 0.818), Cftr (cystic fibrosis transmembrane conductance regulator isoform X1) (*r* = 0.769), and Clca4 (calcium-activated chloride channel regulator 4 precursor) (*r* = 0.783) (*P* < 0.01). However, the serum level of DCA showed negative correlation with three genes, including Gast (gastrin); *r* = −0.769), Atp4b (H (+)/K (+) ATPase subunit beta; *r* = −0.797), and Atp4a (H (+)/K (+) ATPase subunit alpha; *r* = −0.818; *P* < 0.01). The three genes were all involved in the gastric acid secretion pathway, suggesting that DCA might promote GIM *via* inhibiting gastric acid secretion. Furthermore, the bacteria positively correlated with DCA, *Rikenellaceae RC9 gut group*, showed a positive correlation with Slc22a1 (*r* = 0.911; *P* < 0.01) but showed a negative correlation with Pla2g2d (group IID secretory phospholipase A2 precursor; *r* = −0.858; *P* < 0.01). In addition, *Rikenellaceae RC9 gut group* was positively correlated with Fabp1 in fat digestion and absorption (*r* = 0.843; *P* < 0.01) but was negatively correlated with the expression levels of Atp4a and Atp4b in gastric acid secretion (*r* = −0.824, −0.821, *P* < 0.01), suggesting that *Rikenellaceae RC9 gut group* participates in DCA-induced GIM by promoting gastric fat digestion and absorption and inhibiting gastric acid secretion.

Further analysis showed that the bacteria enriched in the model group had negative correlations with the most expression genes in the gastric acid secretion pathway and had positive correlations with the most expression genes in the fat digestion and absorption pathway. For example, the genus with the highest abundance, *Burkholderia–Caballeronia–Paraburkholderia*, was enriched in the model group, positively correlated with Nr1h4 (BA receptor isoform X2) and Fabp1, and negatively correlated with Car2 and Atp4b involved in gastric acid secretion. Another gene, Dgat1, which participates in fat digestion and absorption, was positively correlated with several genera, such as *Sphingomonas, Romboutsia, Ligilactobacillus*, and *Lachnoclostridium*. In the control group, several dominant bacteria (*Bacillus, Rodentibacter*, and *Turicibacter*) had negative correlations with Slc51a and Slc51b, which are involved in bile secretion; in particular, the genus *Turicibacter* was positively correlated with several genes (Gast, Atp4a, and Atp4b) in gastric acid secretion. These results showed that the gastric microbiota plays an important role in maintaining the normal function of the stomach and participates in the development of gastric disorders. Consequently, based on the network data, four signaling pathways (bile secretion, gastric acid secretion, pancreatic secretion, and fat digestion and absorption) played significant roles in the occurrence of GIM. Thus, RT-PCR was used in verifying the expression levels of nine genes (*Fabp1, Cftr, Cdh17, Dgat1, Atp4b, Nr1h4, Slc51a, Gast*, and *Car2*) in the four signaling pathways, and the results were consistent with the results of gene expression profiles (Figure 6B). To confirm the fat digestion and absorption function in GIM tissues, we stained FABP1 and DGAT1 proteins by IHC (Figure 6C). Compared with the control group, the expressions of Fabp1 and Dgat1 in gastric antrum tissue of GIM rats induced by bile reflux were increased.

**Figure 7:**
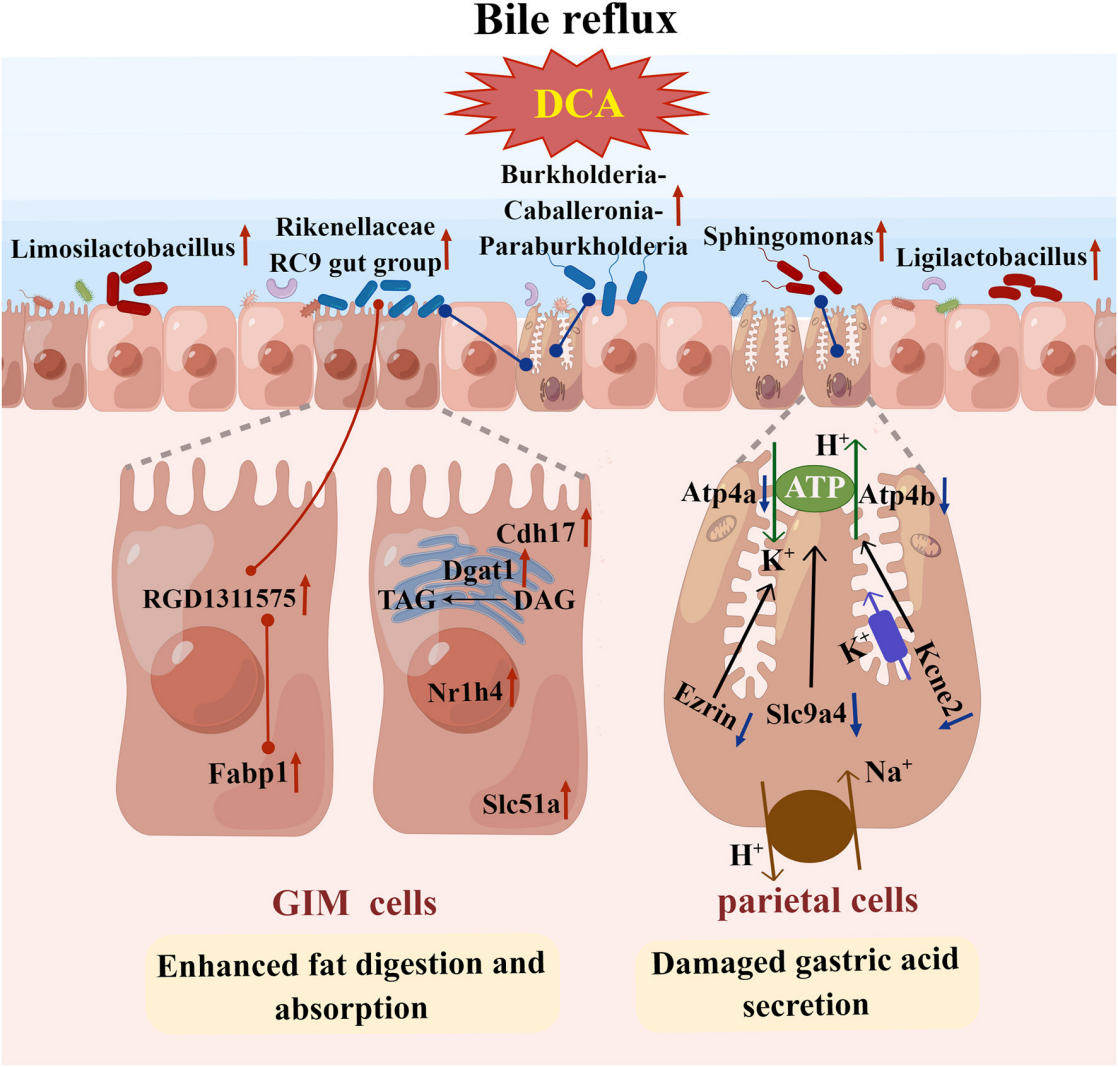
Interaction between gastric microbiota and gene expression profiles during the development of bile acid-induced gastric intestinal metaplasia. When the rat model of gastric intestinal metaplasia was successfully induced by the combination of 2% sodium salicylate and 20 mmol/L sodium deoxycholate for 12 weeks, the expression levels of genes related to gastric acid secretion decreased in the parietal cells, consistent with the clinical symptoms of insufficient gastric acid secretion. Here, we observed marked differences in gastric microbiota, and gene expression profiles in the gastric mucosa induced by DCA-simulative bile reflux. In particular, gastric intestinal metaplasia enhanced the function of fat digestion and absorption, and several characteristic molecules in the intestinal mucosa epithelium appeared in the gastric mucosa, such as FABP1 and RDG1211575. Thus, DCA promoted the proliferation of *Rikenellaceae RC9 gut group* and participated in the transformation of gastric mucosal epithelium to gastric intestinal metaplasia. The red line represents a significant positive correlation, whereas the blue line indicates a significant negative correlation (By Figdraw).

Gastric parietal cells secrete hydrochloric acid to maintain the strong acidic environment in the stomach (pH <2). The strong acid exterminates most food-derived bacteria, facilitates food digestion, and promotes the absorption of minerals, such as phosphate, calcium, and iron. The mucus layer contains rich ${\text{HCO}}_{3}^{-}$-neutralizing hydrion (H^+^) to protect the gastric mucosa against damage caused by the strong acid (Yandrapu and Sarosiek, 2015). Gastric acid secretion depends on H^+^/K^+^-ATPase and various ion transport channels in parietal cells, such as Na^+^/H^+^ exchanger (Slc9a4) and K^+^ channel (Kcnq1 and its subunit Kcne2) (Yuan et al., 2020). Animal experiments found that Slc9a4 knockout can obviously reduce the number of parietal cells in mice, and the function of H^+^/K^+^-ATPase almost disappeared (Gawenis et al., 2005). Meanwhile, Kcne2 knockout can promote the proliferation of non-acid secretory cells in mouse gastric glands, and gastric acid secretory cells cannot respond to histamine stimulation, suggesting that the *Kcne2* gene is involved in the regulation of gastric acid secretion (Roepke et al., 2006). In addition, during gastric acid secretion, parietal cells undergo multiple morphological transformations, including the expansion of intracellular canaliculi, increase in the number and length of the apical microvilli, entry of H^+^/K^+^-ATPase into the secretory canaliculus from cytoplasmic vesicles, and fusion with an apical membrane (Sachs et al., 2007). Knocking out ezrin, as an actin-binding protein, can inhibit the microtubule of H^+^/K^+^-ATPase in parietal cells from fusing with the apical membrane and promote defective gastric acid secretion (Tamura et al., 2005). Ezrin's Ser-66 and Thr-567 are phosphorylated by PKA (protein kinase A), phosphorylated ezrin combines with MST4 (protein kinase domain containing protein) to promote the insertion of tubular vesicles carrying H^+^/K^+^-ATPase into the cell membrane, and gastric acid secretion is promoted (Jiang et al., 2015; Yuan et al., 2017). Thus, ezrin plays a key role in regulating the cycle of H^+^/K^+^-ATPase. In the present study, DCA-induced GIM obviously reduced the expression level of ezrin, and this mechanism may be a key factor in impaired gastric acid function. When GIM occurs, the enhanced fat digestion and absorption function of the gastric mucosa is closely linked to the increased expression of several GIM-related molecules, such as Dgat1, Fabp1, and Slc51a (Figure 7). These results suggested that the interaction between gastric microbiota and reflux BAs is involved in the occurrence of GIM, providing a new perspective for exploring the biological mechanism of GIM development.

## 4. Discussion

Bile reflux, also known as duodenogastric reflux, refers to the backflow of duodenal contents (including bile, pancreatic juice, and duodenal juice) into the stomach. A substantial amount of epidemiological evidence has shown that bile reflux is an independent risk factor for CAG, IM, and GC (Matsuhisa et al., 2013; Zhang et al., 2021a). Obvious alteration of the microbiota in the digestive tract is closely linked to GC development and its precancerous lesions; dysbacteriosis in the stomach is correlated with gastric mucosal lesions (Lu et al., 2022). The reflux of alkaline bile into the stomach can obviously change the gastric environment and shape the composition of gastric microbiota. For example, Huang et al. (2022) observed 92 patients with CG and found that patients with CAG accompanied by bile reflux had higher microbial diversity and richness than those in non-atrophic gastritis patients without bile reflux (Huang et al., 2022). However, our results showed induced richness of gastric microbiota in the GIM rats. The possible reasons are the sterile diet and the antibacterial properties of DCA. For instance, an *in vitro* experiment showed that CA and DCA can inhibit bacterial proliferation, such as the Gram-positive bacteria *Clostridium perfringens* and Gram-negative bacteria *Lactobacilli* and *Bifidobacteria* (Tian et al., 2020). Therefore, BAs can deeply affect the community of gastric microbiota and participate in the occurrence and development of gastric mucosal lesions.

The present study found that several dominant bacteria were enriched in the gastric mucosa of GIM rats, such as *Limosilactobacillus, Ligilactobacillus, Burkholderia–Caballeronia–Paraburkholderia, Ligilactobacillus, Romboutsia*, and *Bradyrhizobium*. Delgado et al. (2013) reported that *Lactobacillus* was a dominant genus in gastric mucosa and gastric juice of healthy people in Spain (Delgado et al., 2013), Gantuya et al. (2020) reported its relative abundance in the gastric mucosa of Hp-negative GC patients ranging from 35.2 to 97.0% (Gantuya et al., 2020). He et al. (2022a) and Hsieh et al. (2018) found that the genus *Lactobacillus* gradually increased with the cascade process of gastric carcinogenesis and was the dominant genus in gastric mucosa of GC patients. The latest literature found that DCA can induce GIM and dysplasia in the INS-GAS mice and is accompanied by an increasing abundance of gastric *Lactobacillus* (Jin et al., 2022). These results indicated that the high abundance of *Lactobacillus* is closely related to gastric mucosal atrophy, GIM, and GC. Lactic acid-producing *Lactobacillus* may have dual effects. On the one hand, lactic acid can reduce the pH value of the gastrointestinal tract, antagonize pathogenic bacteria, such as *Hp*, in gastric mucosa, and play a beneficial role; on the other hand, lactic acid can acidify the gastric mucus layer (Carr et al., 2002), inhibit the secretion of gastrin and gastric acid by gastrin cells of gastric antrum (Myllyluoma et al., 2007), promote gastric mucosal atrophy, and exert the damaging effect of the gastric mucosa. Here, the abundance of the genus *Burkholderia–Caballeronia–Paraburkholderia* increased in the gastric mucosa of the GIM rats, consistent with two previous studies. Increased bacterium was observed in the gastric mucosa of GIM patients (Hsieh et al., 2018) and increased steadily with the advancement of atrophic gastritis to dysplasia in Hp-negative patients (Sun et al., 2022). Zhang et al. (2021b) found that gastric *Romboutsia* was enriched in the patients with gastric intraepithelial neoplasia (Zhang et al., 2021b); this result was consistent with the results of the present study. The genus *Bradyrhizobium* is a well-known symbiotic bacterium in the nodules of leguminous plants and can reduce nitrogen into nitrogenous nutrients. Park et al. (2019) observed that *Rhizobiales* was commonly distributed in the gastric mucosa of GIM patients, and its relative abundance gradually increased with decreasing abundance of *Helicobacter*, indicating a competitive antagonistic relationship between *Romboutsia* and *Helicobacter*. Therefore, the alteration of the gastric microbiota in these GIM rats was mainly related to the maintenance of the gastric acid environment, transformation of nitrogen substances, and commensal relationship. In addition, the abundances of *Bacillus* and *Streptococcus* significantly decreased in the model rats, suggesting their protective effects against GIM development. The bacterial spores of the genus *Bacillus* (*Bacillus subtilis* and *Bacillus coagulans*) have been widely used as oral probiotic preparations to improve gut microbial balance and enhance immune capacity (Cutting, 2011). Zhang et al. (2020) investigated the effect of the oral administration of probiotics on patients with *Hp* infection and found that the combination of *Clostridium butyricum* and *Bacillus coagulans* achieved higher eradication rates (26%) than the single *Clostridium butyricum* (18%) or *Bacillus coagulans* (20%). This result indicated that *Bacillus* has an antagonistic effect on *Hp* and blocks gastric mucosal lesions. The similar protective effects of *Bacillus* and *Streptococcus* were observed in functional dyspepsia (Wauters et al., 2021), and an *in vitro* experiment presented that *Streptococcus thermophilus* CRL1190 strain can inhibit *Hp* adhesion and attenuate inflammatory response in human gastric epithelial cells (Marcial et al., 2017). Furthermore, *in vitro* and *in vivo* studies have shown that *Streptococcus thermophilus* possesses healthy probiotic properties, such as immunomodulatory and antioxidant functions and colonization resistance (Ito et al., 2008; Ogita et al., 2011). These results demonstrated that supplementation with *Bacillus* and *Lactococcus* is a probiotic strategy for preventing and treating bile reflux-inducing GIM.

Based on the predictive KEGG function of gastric microbiota, two enhanced signaling pathways deserve attention in GIM rats: p53 and RIG-I-like receptor signaling pathways. Sasaki et al. (2021) found that weak acidic BAs can promote the precancerous lesions of hypopharyngeal mucosa. In particular, the presence of DCA can promote the overexpression of cancer-promoting genes, including epidermal growth factor receptor, *Wnt5a*, and *Rela*, which rely on DNA/RNA oxidative damage and p53 signaling pathway. The RIG-I-like receptor is known for its innate antiviral immunity (Onomoto et al., 2021). Viral infection can induce immediate early NF-κB activation and triggers the rapid transcription of the BA transporter SLCO1B2 (solute carrier organic anion transporter family member 1B2) and BA-synthesis-rate-limiting enzymes, including CYP7A1 (cholesterol 7α-hydroxylase), CYP7B1 (cytochrome P450 family member 7B1), and CYP27A1 (cytochrome P450 family member 27A). Then, accumulated BAs appear in hepatocytes and monocytes and activate the SRC kinase and mediated RIG-I signaling pathway to undertake antiviral immunity *via* the TGR5–GRK–β-arrestin axis (Hu et al., 2019). The results suggested that bile reflux can induce gastric gene mutation and chronic inflammation and promote the development of GIM and GC. Here, we found that the primary and secondary BA biosynthesis pathways were enriched in the DCA-induced GIM rats. Substantial epidemiological evidence has shown that the gut microbiota can facilitate the conversion of primary BAs into secondary BAs, and the process is dependent on many kinds of bacterial enzyme catalysis processes, including deconjugation, dehydroxylation, oxidation, and epimerization (Guzior and Quinn, 2021). For instance, bile salt hydrolase, which is widely expressed in the bacterial genera *Clostridium, Bacteroides, Lactobacillus, Bifidobacterium*, and *Enterococcus*, can rapidly remove glycine or taurine residues from primary BAs (Kiriyama and Nochi, 2021). This study found that *Bacteroides, Limosilactobacillus*, and *Blautia* were enriched in the GIM group. Thus, the gastric microbiota might participate in the transformation between primary BAs and secondary BAs when bile reflux occurs, and the interaction between BAs and gastric microbiota plays an important role in the occurrence of GIM.

An advanced analysis of the 23 detected serum BAs showed that the four conjugated BAs (TCA, GCA, TDCA, and GDCA) significantly increased. Zhao et al. found that the four conjugated BAs (GCA, GCDCA, TCA, and TCDCA) significantly increased in the gastric juice of gastritis patients with bile reflux (Zhao et al., 2020), suggesting that the levels of BAs in the serum may be consistent with those in the gastric juice. In particular, GCA and TCA might be the key BAs that induce the occurrence of GIM. Wang et al. (2022) investigated the relationship between gastric microbiota and bile reflux. A clinical study showed that an increase in conjugated BAs in the gastric juice is associated with the elevated abundance of *Prevotella melanogenesis* in patients with gastritis and bile reflux and patients with GC. Combined gavaged TDCA and *Prevotella melanogenesis* can promote gastric inflammation. Finally, the *in vitro* experiment confirmed that TDCA and LPS can promote the proliferation of normal gastric epithelial cells (GES-1) by activating the IL-6/JAK1/STAT3 pathway (Wang et al., 2022). Yang et al. (2022b) observed a positive correlation between gastric DCA and *Lachnospiraceae NK4A136 group* in patients with CG and bile reflux (Yang et al., 2022b). The present study observed another positive correlation between DCA and *Lachnospiraceae UCG-006* belonging to the family *Lachnospiraceae*. These results suggested that *Lachnospiraceae* bacteria might be the main bacteria responding to bile reflux in the development of GIM, and the interaction between BAs and gastric microbiota plays a potential role in the inflammatory proliferation of gastric mucosal cells.

BAs participate in the development of digestive system tumors through blood circulation. Li et al. designed a case–control study in a Chinese population and found that the serum total BA >10 μmol/L can be a diagnostic threshold for the risk of gastrointestinal cancer, especially for the risk of esophageal cancer and GC (Li et al., 2022b). The latest clinical research (Pan et al., 2022) presented clearly promoted conjugated and unconjugated BAs in the sera of patients with GC, again showing that serum BAs participate in the development of gastric carcinogenesis apart from GIM. The present transcriptome of the gastric mucosa showed that the overexpression levels of GIM-related genes were consistent with those in the previous reports, such as *Cdx2, Nr1h4*, and *Cdh17*. However, the expression level of Sox2 significantly decreased. Yuan et al. found that DCA can inhibit the expression of SOX2 in human GC cells AGS, AZ-521, and MKN45 and promote CDX2 expression in human gastric epithelial cells GES-1 (Yuan et al., 2019). Yu et al. (2019) found that CDCA and DCA can activate the FXR/NF-κB pathway and promote CDX2 and MUC2 in human gastric epithelial cells GES-1. Ko et al. (2005) found that the overexpression of CDX2 has a significant positive correlation with CDH17 in the tissues of gastric adenocarcinoma. Lee et al. (2010) found overexpressed CDH17 in the tissues of GC and spasmolytic polypeptide-expressing metaplasia. The data confirmed the molecular basis for the reliability of the present GIM rats.

Notably, based on the gene expression profiles of gastric mucosa, the four key signaling pathways were the focus of discussion, including gastric acid secretion, bile secretion, pancreatic secretion, and fat digestion and absorption. Based on the microarray datasets of the GIM/GC tissues, the upregulated genes were enriched in two physiological functions (fat digestion and absorption, and metabolic pathways of protein, amino acid, and fat), whereas the downregulated genes were enriched in gastric acid secretion (Pang et al., 2021). Under normal conditions, the small intestine rather than the stomach digests and absorbs fat (Ko et al., 2020). However, the latest prospective lipidomics study showed that 11 plasma lipids are significantly inversely correlated with the risk of gastric lesion progression and GC occurrence, and the pathway enrichment analysis showed that the four most related signaling pathways, such as arachidonic acid metabolism, α-linolenic acid metabolism, linoleic acid metabolism, and glycerophospholipid metabolism, are associated with GC and gastric lesion progression (Liu et al., 2022b), suggesting that abnormal lipid metabolism is related to gastric carcinogenesis. The present study found two lipid-metabolism-related genes (*DGAT1* and *FABP1*) overexpressed in the gastric mucosae of GIM rats. DGAT1 is the key enzyme that catalyzes the conversion of diacylglycerol to triacylglycerol and plays an important role in intestinal fat absorption, lipoprotein assembly, adipose tissue formation, and other physiological processes (Cases et al., 1998). When the *DGAT1* gene was knocked out, a variety of fat metabolism disorders started to occur, such as increases in obesity resistance and energy consumption in mice (Smith et al., 2000) and decreased fatty acid synthesis and increased oxidative decomposition of fatty acids in the liver (Villanueva et al., 2009). A meta-analysis based on the TCGA database revealed that the overexpressed DGAT1 in GC tissues and tumor-infiltrating immune cells was associated with poor pathological differentiation and poor prognosis (He et al., 2021). FABP1 is an intracellular protein that binds long-chain fatty acids, is widely expressed in the liver and intestinal epithelium, and is involved in adipogenesis and lipid metabolism (Wang et al., 2015). The latest integrated single-cell RNA-sequencing analysis revealed that the overexpression of FABP1 is a novel biomarker of poor prognosis in patients with GC and is involved in the peroxisome proliferator-activated receptors (PPAR) signaling pathway, hormone-sensitive lipase-mediated triacylglycerol hydrolysis, and fat digestion and absorption (Yang et al., 2022a). Evidence has shown that fatty acids are not only the key energy sources for GC cells but also are crucial substances for membrane biosynthesis in rapidly proliferating GC cells (Li et al., 2022a). We observed upregulated apolipoprotein genes (*Apoa1* and *Apoa4*) in the gastric mucosae of DCA-induced GIM rats. This finding indicated that bile reflux enhances gastric fat digestion and absorption function.

Notably, a correlation network, “DCA–*Rikenellaceae RC9 gut group*–RGD1311575–Fabp1,” was found. Wang et al. found that a high-fat diet enriched the gut *Rikenellaceae RC9 gut group* in C57BL/6J mice (Wang et al., 2020a). Yang et al. (2022c) found abundant *Rikenellaceae RC9 gut group* in the proximal GC tissue and was positively correlated with the tissue N-acetylneuraminic acid, which is a sialic acid and plays a crucial role in tumor immune evasion and immune-permissive tumor microenvironment (Büll et al., 2018). Overall, the correlation network “DCA–*Rikenellaceae RC9 gut group*–RGD1311575/Fabp1” created a cancer-promoting microenvironment in the stomach during the development of bile reflux-related GIM. Therefore, our future study will focus on bridging the gap between bile reflux-induced GIM and enhanced fat digestion and absorption function and is expected to provide novel intervention strategies for preventing and treating precancerous lesions and GC.

## Data availability statement

The datasets presented in this study can be found in online repositories. The raw reads of gastric mucosa microbiota and gastric mucosa transcriptome were deposited into the NCBI Sequence Read Archive (SRA) database, and the Accession Numbers are SRP423819 and SRP424012, respectively.

## Ethics statement

The animal study was reviewed and approved by the Animal Ethics Committee of Nanjing University of Chinese Medicine.

## Author contributions

JZ and CC contributed to the conception and design of the study. ZX and SW conducted experiments and maintained the animals. LX and YC participated in the sample collection. ZX, YC, and SW analyzed all the animal data. JW, KB, and YM provided technical support to the animal experiment. ZX, LX, and YC drafted the manuscript. JZ and CC critically reviewed and revised the manuscript. ZX and LX contributed equally to this manuscript. All authors contributed to the article and approved the submitted version.
