# Supplementary material for: Alteration of gastric microbiota and transcriptome in a rat with gastric intestinal metaplasia induced by deoxycholic acid
Source: Front Microbiol. 2023 May 3;14:1160821. doi: 10.3389/fmicb.2023.1160821 (PMC10188980; doi:10.3389/fmicb.2023.1160821)
Supplement: Supplementary file 1 [file Data_Sheet_1.docx]

Supplementary Material

# Supplementary Figures and Tables

## Supplementary Figures


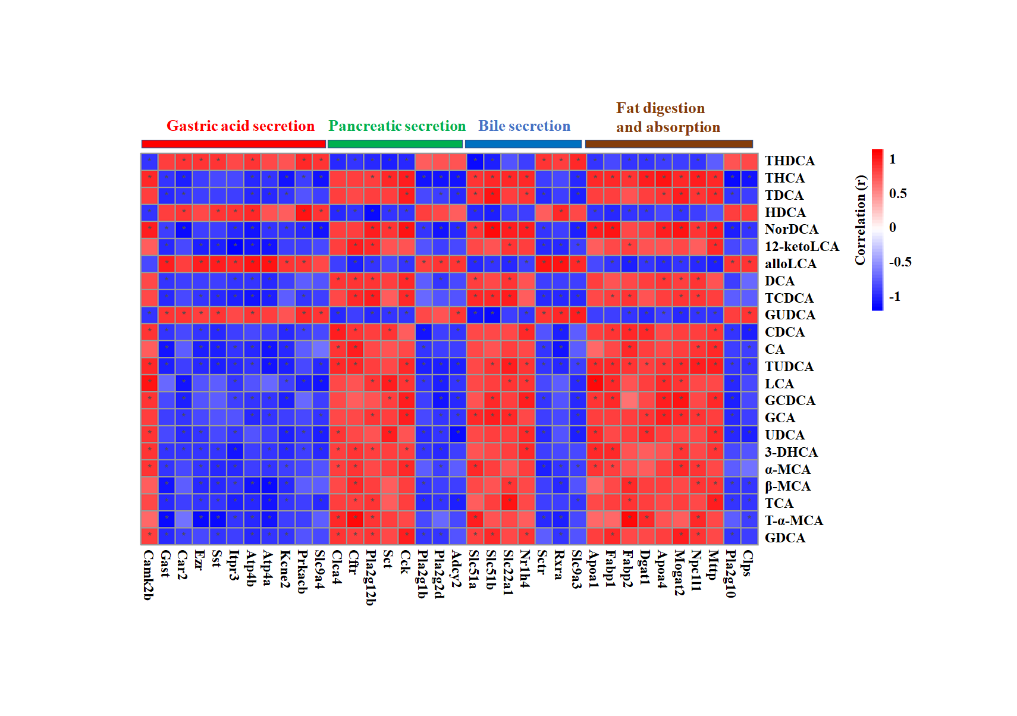


**Supplementary Figure 1.** Correlation heatmaps between DEGs and different serum bile acids in the occurrence of GIM. In the total samples, Spearman correlation analysis was used in conducting a heatmap based on the gastric expression levels of genes in the four signaling pathways and serum bile acids. The red squares represent significant positive correlations, and the blue squares represent significant negative correlations. The asterisks mean significant correlations with an absolute correlation coefficient (r) greater than 0.75 and *P* value less than 0.05.


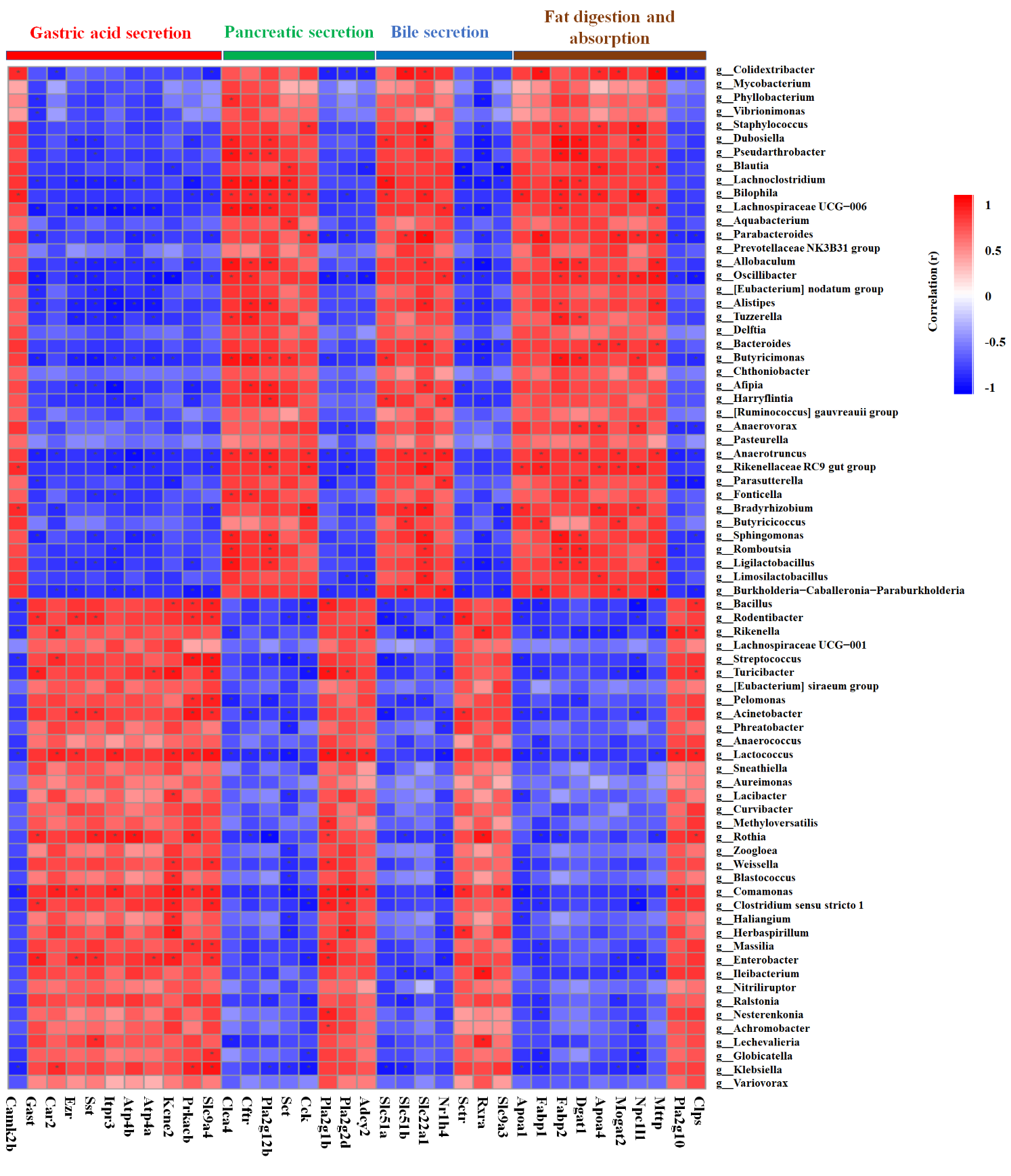


**Supplementary Figure 2.** Correlation heatmaps between DEGs and different bacteria in the occurrence of GIM. In the total samples, Spearman correlation analysis was used in constructing a heatmap based on the gastric expression levels of genes in the four signaling pathways and different bacterial genera. The red squares represent significant positive correlations, and the blue squares represent significant negative correlations. The asterisks mean significant correlations with the absolute correlation coefficient (r) greater than 0.80 and *P* value less than 0.05.

**Table S1. Sequence of primers used in the study**

| **Gene name** | **Forward sequence** | **Reverse sequence** |
| --- | --- | --- |
| β-actin | CTGGCTGGCCGGGACCTGACA | ATTGCCGATAGTGATGACCTG |
| Fabp1 | GCGATGGGTCTGCCTGAG | CACGGACTTTATGCCTTTGAA |
| Cftr | ACAGGGAGTGAGGAAGACAAGGAT | TCAAAGCTGCATTAAAAGGGAGTA |
| Cdh17 | GGGGCCCACTATTCCTTA | TGTCGGTGGGTTGTCATTA |
| Dgat1 | GGCCAGCCCTCCCTAACCT | TCATCCTCAGCAGCCTCTACAACC |
| Atp4b | AAAGCCCCGGAAGGACATTGAACC | TTGAGGAACTTTGCCGCCACCAGA |
| Nr1h4 | ACCCCGCTCCTCTGTGA | TTCCCGAAGCCTTGTAAAAT |
| Slc51a | GCCCTGCAGCCCTCCATT | GCGGTAAACAAGCCTCAT |
| Gast | CAATGGGGCCCTGGAACA | GCTGGGGGTGGCTGGAGAT |
| Car2 | AGGGGGAGGCTGAAGAACTGA | ACCAAGGCGGAACTGTGCT |
